# Probing the character of ultra-fast dislocations

**DOI:** 10.1038/srep16892

**Published:** 2015-11-23

**Authors:** C. J. Ruestes, E. M. Bringa, R. E. Rudd, B. A. Remington, T. P. Remington, M. A. Meyers

**Affiliations:** 1 Facultad de Ciencias Exactas y Naturales, Univ. Nac. de Cuyo, Mendoza 5500, Argentina; 2 University of California, San Diego, La Jolla, CA 92093, USA; 3 Lawrence Livermore National Laboratory, Livermore, CA 94550, USA

## Abstract

Plasticity is often controlled by dislocation motion, which was first measured for low pressure, low strain rate conditions decades ago. However, many applications require knowledge of dislocation motion at high stress conditions where the data are sparse, and come from indirect measurements dominated by the effect of dislocation density rather than velocity. Here we make predictions based on atomistic simulations that form the basis for a new approach to measure dislocation velocities directly at extreme conditions using three steps: create prismatic dislocation loops in a near-surface region using nanoindentation, drive the dislocations with a shockwave, and use electron microscopy to determine how far the dislocations moved and thus their velocity at extreme stress and strain rate conditions. We report on atomistic simulations of tantalum that make detailed predictions of dislocation flow, and find that the approach is feasible and can uncover an exciting range of phenomena, such as transonic dislocations and a novel form of loop stretching. The simulated configuration enables a new class of experiments to probe average dislocation velocity at very high applied shear stress.

The motion of defects in the crystal lattice is essential to our understanding of material strength and plastic deformation. Across a broad range of conditions, dislocations make the most important contribution. Dislocations move in response to shear stress, and for a given density of dislocations, the faster they move, the higher the strain rate is.

At low stresses, a variety of tools is available to study the motion of dislocations: etch pits^1^, *in-situ* transmission electron microscopy (TEM)^2^, inelastic acoustic response^3^, and computer modeling^4^.

Under extreme conditions, dislocation motion is still essential, but our understanding of that motion is remarkably incomplete. The collected information is sparse, and from indirect measurements like perturbation of growth in Rayleigh-Taylor experiments^5^, or lateral lattice relaxation measured with x-ray diffraction^6^,^7^,^8^. These experiments are dominated by the effect of dislocation density rather than velocity. At high stresses, dislocations move so rapidly that no experimental technique has been available to study them directly. Atomistic models provide tremendous detail about dislocation flow mechanisms. For instance, computer modeling has made predictions of transonic dislocation motion^9^, extraordinary debris generation^10^, and homogeneous nucleation^11^, but these enticing predictions have not been tested experimentally directly.

Molecular dynamics (MD) simulations have already been used to simulate the motion of a single “straight” dislocation at high stresses^9^,^10^, but here we study dislocation-loop motion at high stress, and qualitatively new mechanisms arise. In this Letter we emphasize transonic dislocations, i.e., dislocations that move faster than the shear wave velocity. From the late 1940s until the late 1960s, it was assumed that dislocations were not able to move above the shear wave velocity because linear elasticity theory predicts the need of an infinite energy to move at such velocity^12^. Supersonic dislocations would move with velocities above the longitudinal sound velocity C_L_, and transonic dislocations would move with velocities above the transverse sound velocity C_T_. Since the seminal work by Frenkel and Kontorova in 1938^13^, Frank and van der Merwe in 1949^14^ and Weertman in 1967^15^, the issue of transonic and supersonic dislocations has brought a long, yet unsolved, debate on the possibility of dislocations moving steadily above the limit given by the longitudinal wave sound speed. More recently, in computational studies, Gumbsch and Gao^9^ showed the stable motion of edge dislocations in tungsten with velocities in the transonic and supersonic regimes. They stated that, for these velocities to be achieved, dislocations had to be created already with high velocities under high shear stresses. Their work was important because it demonstrated for the first time the existence of stable dislocation motion above the shear wave velocity. This contribution was followed by that of Jin *et al.*^16^, who also reached those regimes but from stationary configurations, showing for the first time that the requirement of infinite energy^12^ is more of a pathology in the mathematical analysis than a real physical limit because discrete atoms are not subject to the singularities found in the continuum theory. In 2004, Vandersall and Wirth^17^ reported unstable dislocation motion in the transonic regime when studying aluminum, also demonstrating that twinning is the preferred mode of deformation under high stresses. In the late 2009, Tsuzuki *et al.*^18^ reported unstable supersonic dislocations when studying copper under high strain shear with MD, claiming that transonic motion can be stable depending on the applied shear stress and on the dependence of dislocation velocity on the applied shear strain. Tang *et al.*^19^ calculated dislocation velocities from MD simulations of homogeneous compression of nanovoided tantalum and obtained subsonic values in the range of 680 m s^−1^ to 1020 m s^−1^ for strain rates in the range 10^8^ s^−1^ − 10^9^ s^−1^. All of these aforementioned studies are computational: there is no direct experimental proof of the existence of transonic or supersonic dislocation motion. Those studies concentrated either on pure edge dislocations or on edge components of shear loops, while the focus of this letter is on the motion of prismatic loops.

Experimental conditions which could lead to supersonic dislocations can be produced by shock compression. In particular, the response of materials to laser-driven shock compression has been the subject of tremendous research efforts over the past ~10 years. The recent experimental breakthroughs with *in situ* white-light x-ray Laue diffraction^20^ and on ultrafast powder diffraction^8^ have allowed access to quantitative information of lattice distortions and strength in bcc Ta and fcc Cu^7^ under laser-driven shock compression. However, these experiments measure the combined effect of dislocation density and dislocation velocity on the plastic strain, and rely on dislocation velocity estimates to extract dislocation densities. In addition, constitutive models used to predict mechanical behavior of materials under extreme loading conditions increasingly rely on simulated dislocation velocities, given the lack of experimental data^21^. As a result, the detailed mechanisms of plasticity under those extreme-loading conditions are not only poorly understood, but this gap leaves advanced constitutive models on an uncertain foundation.

Our simulations demonstrate that nanoindentation may be used to create a well-defined dislocation source near the surface of a defect free single crystal of metal^22^,^23^, in this case the body-centered cubic (bcc) metal tantalum, as a model bcc metal for which significant high pressure data exists. Nanoindentation in a bcc metal is dominated by dislocations, and the high Peierls stress stabilizes the dislocation configuration. The indented specimen is then shock-loaded, and dislocations move very rapidly in response to the high shear stress in the shocked state, from the near-surface region into the bulk. Again, the high Peierls stress stabilizes the dislocation positions as the shear stress drops during the release from the shocked state. The distance that the dislocations travel may be determined by TEM, and the loading stress inferred from surface velocity measurements. Together they constitute a direct measure of dislocation mobility at high stress.

In our simulations, we aim to predict, and ultimately experimentally infer, dislocation velocities resulting from ultrahigh applied shear stresses. The first step is the presence of well-characterized dislocation loops. Controlled introduction of dislocation loops could be achieved in several ways, and here we focus on nanoindentation, where dislocation density and spatial extent can be measured from TEM imaging or could be estimated indirectly by the size of pile-ups or the number of pop-ins in the loading curve up to modest penetrations. The second step is to drive those existing dislocations using a short duration laser-induced shock wave. Finally, measurements of the evolution of the plastically deformed region allow for estimations of dislocation velocities. MD simulations are presented here to demonstrate both the dislocation distributions from the nanoindentation and their evolution due to a strong, intense shock (Shock pressure in the range of 10 to 100 GPa and shock duration of 0.1 to 10 ns).

## Results

**Figure 1:**
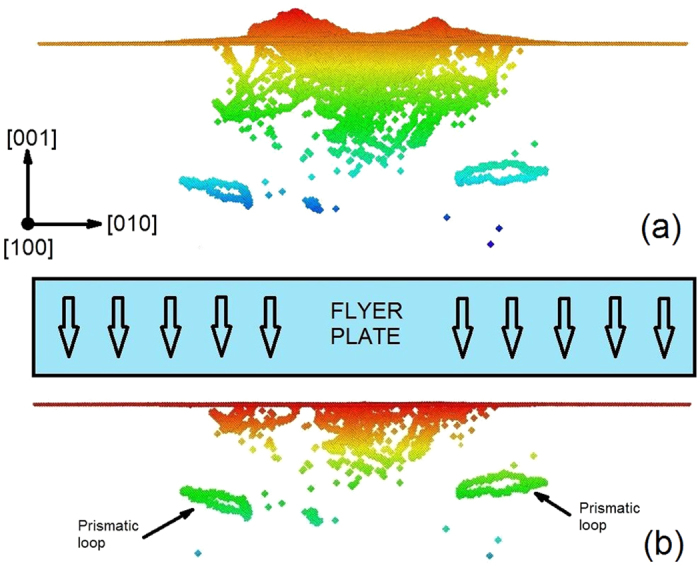
(**a**) Microstructure left after nanoindentation and unloading, including surface pile-ups; (**b**) after removal of surface defects, indentation pile-up and imprint on the target, the simulation setup was completed by adding a flyer plate to impact on the sample surface. Qualitative color scale indicates depth into the sample, with red corresponding to surface atoms. Only atoms at defects are shown.

**Figure 2:**
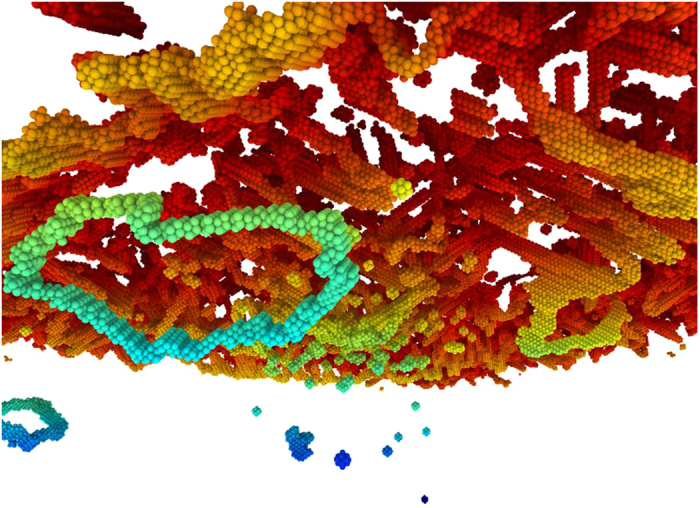
Snapshot of the target, 2 ps after being hit by the flyer plate at 1250 m s^−1^. Color scale indicates depth, with red corresponding to the surface. There is profuse generation of dislocation loops from the surface (red and orange), together with motion of the pre-existing prismatic loops (green-blue). A few point-defect clusters are also seen in green-blue.

Nanoindentation produces a limited number of defects confined to a volume close to the indentation imprint, as shown in Fig. 1(a). The nucleated defects include prismatic loops. To assess the effect of the shock loading on the prismatic loops and, in an effort to guarantee a flat shock front, the indentation imprint and material within a few nanometers depth was removed prior to shock loading (Fig. 1b). Shock-induced strain is accommodated by motion of pre-existing dislocations and the possible nucleation of new dislocations. We consider the case of a shock produced by plate impact, although other shock sources are possible and may be preferred in practice. The interface between the flyer plate and the sample acts as a very efficient source of new dislocations, as shown in Fig. 2. This process produces “shear” dislocation loops attached to the surface, whose motion can be tracked to obtain their velocity, but only for relatively short times due to a high dislocation density, leading to rapid junction formation which slows them down. The basic difference between shear and prismatic loops is that the Burgers vector is in the plane of the loop in the former and perpendicular to it in the latter. We note that similar dislocations are nucleated into the flyer plate and propagate there in the opposite direction. As expected, shear loops evolve on {110} and {112} planes, sharing the same <111> Burgers vector, with the edge component driven into the material, and the screw component pinned to the surface.

**Figure 3:**
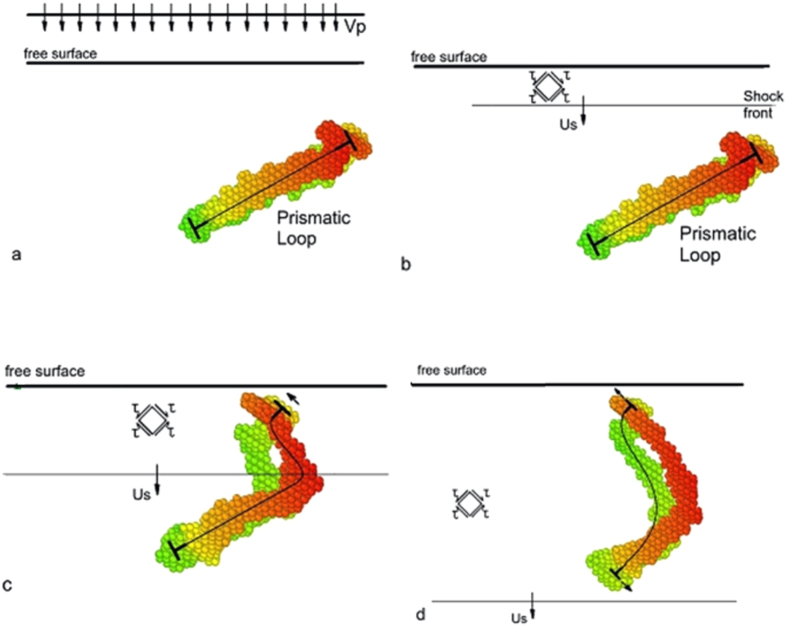
Schematic sequence showing movement of a single prismatic loop (solid lines) from a side view under shock compression produced by impact of a flyer plate with velocity V_F_; (a) flyer plate approaching target containing prismatic loop; (b) shock front penetrating target and leading to large shear stresses; (c) shock front interacting with top portion of prismatic loop; (d) movement of top and bottom portions of prismatic loop in opposite directions. MD snapshots corresponding to the schematic representation are also shown. Color represents depth towards the page, with red closer to the observer. (**a**) Existing prismatic loop from nanoindentation; (**b**) shock propagating towards the loop; (**c**) upper portion of the loop moving towards the surface; (**d**) lower portion of the loop moving towards the back surface.

Pre-existing prismatic loops (Fig. 1b) could have been expected to glide away from the surface under the shock-induced stress. The scenario, however, is more complex, and prismatic loops accommodate the applied loading by stretching in opposite directions depending on the sign of the several edge components that constitute them, as shown in Fig. 3. A positive dislocation will move in a direction opposite to a negative dislocation under the same applied shear. This explains the observed behavior because the prismatic loop is composed of edge dislocations with the same Burgers vector but with opposite line directions on opposite sides of the loop (Fig. 3a).

**Figure 4:**
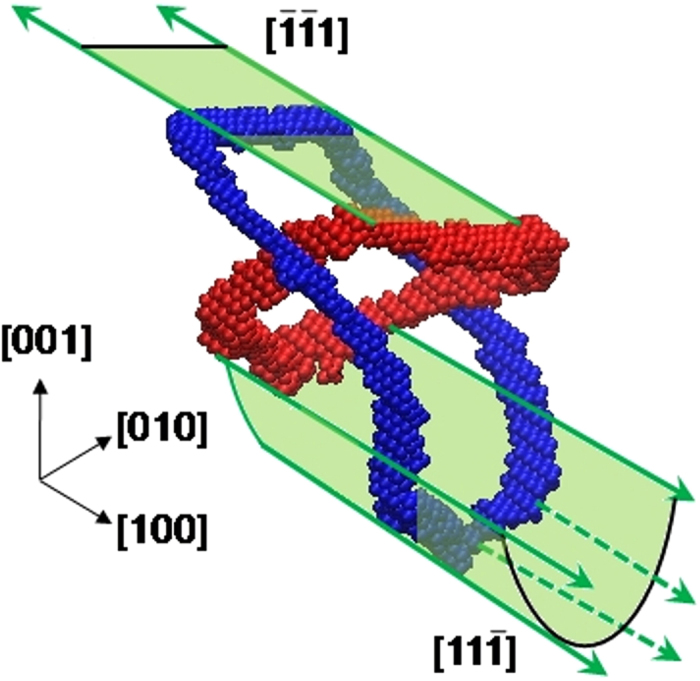
Initial configuration of a pre-existing prismatic loop (red); and configuration of the prismatic loop after being driven by the shock front during 2 ps (blue), showing significant stretching. The upper portion of the loop consists of a single edge component that glides in a single plane as shear stresses actuate. The lower portion of the loop consist of several edge components in a bowed arrangement, they collectively glide in a single plane each, preserving the bowed shape. The motion, growth and shape change of the prismatic loop is a complex three-dimensional process. We focus on the edge components of the loop, each gliding on a plane along a <111> direction, hence studying the process as an effective one-dimensional motion.

As the flyer impacts the target, a compressive shock-wave travels into the target imposing a compressive stress state that can be decomposed into shear components (Fig. 3b). As the shock-wave proceeds through the target, increasingly more sections of the prismatic loop are subjected to shear stresses and, as a consequence, the positive components are displaced in one direction and the negative components in the opposite direction, resulting in the stretching of the prismatic loop, and increasing the dislocation length in this process (Fig. 3c,d). Details of the stretching can be seen in Fig. 4, which shows how the opposite sides of the prismatic loop move in opposite directions while the lateral sides are stationary because the resolved shear stresses acting upon them are nearly zero. This mechanism is quite different from the mechanism operating during nanoindentation. In the planar shock wave the shear stress is roughly constant along the transverse directions, so opposite sides of the loop experience opposite Peach-Koehler forces and the loop is stretched. The morphology of the loops is significant for the experiments. In a quest for proof of extraordinary dislocations (transonic or supersonic), having a long straight screw dislocation in the shock direction might be a signature that the leading edge moved the loop’s length during shock loading.

**Figure 5:**
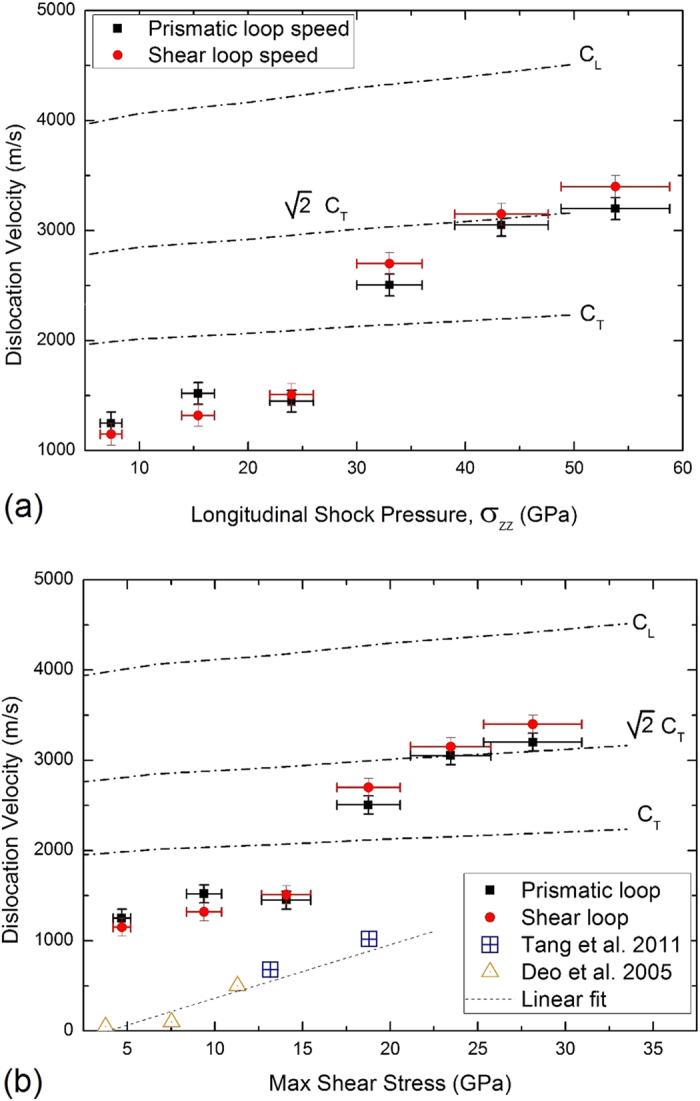
Dislocation velocities as a function of (a) shock-wave pressure, and (b) maximum shear stress. Dislocations reach the transonic regime as the shock-wave pressure approaches a threshold value of 35 GPa, corresponding to a maximum shear stress of 17 GPa.

The velocity of these stretching dislocations was also monitored. For both shear and prismatic loops, velocities exceeding the shear wave speed were found in the simulations, as can be seen in Fig. 5. To properly address different velocity regimes four distinctive velocity regimes were identified^16^,^17^,^18^: a subsonic regime (v < C_T_), a first transonic regime (C_T_ < v < 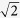
C_T_), a second transonic regime (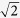
C_T _< v < C_L_), and supersonic regime (v > C_L_). 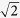
C_T_ is often referred as the intersonic/transonic barrier and is consistent with Eshelby’s continuum formulation for a radiation-free dislocation state in isotropic systems^12^. Considering the formulation from linear elastic theory, dislocations are impeded from moving at speeds above the velocity of the material’s elastic shear waves due to the Lorentz contraction of the dislocation strain field: the energy required becomes infinite. Also, since the postulation of a stable radiation-free state at 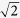
C_T_, several authors^9^,^10^,^16^,^17^ have used this barrier to identify dislocation velocity regimes within the regime between the transverse wave speed and the longitudinal wave speed. Up to a pressure of 30 GPa (and a corresponding maximum shear stress of 17 GPa) the velocities of the dislocations are qualitatively consistent with earlier results by Deo *et al.*^24^ and Tang *et al.*^19^ in the sense that they also fall in the subsonic regime. These results are shown in Fig. 5b. The velocities do not exceed the limit associated with Eshelby’s radiation-free state definition^12^.

TEM studies of nanoindented Ta have been recently presented^22^, and new results are included in the Supplementary Material. In experiments, indentation-generated loops will be significantly larger, and thus their edge component will be more closely related to a straight dislocation. Therefore, an important question to address is whether a loop exhibits the same mobility as a long straight edge dislocation. Due to the large mobility of edge dislocations and the lower mobility of screw dislocations by kink-pair nucleation, most studies of mobility for bcc metals are for screw dislocations only, with the exception of the studies by Bacon, Osetsky and co-workers^25^. For our simulations, following the procedure employed by Marian *et al*. in their Fe study^10^, the phonon-drag coefficient, B_ph_, can be calculated from the slope of the velocity-stress plot of Fig. 5b, 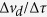. The resulting phonon-drag coefficients are ~7 × 10^−3^ Pa s for the subsonic regime, and ~5 × 10^−3^ Pa s for the transonic regime. A detailed analysis of possible diffusional processes affecting loop motion is also included in the Supplementary Material.

## Discussion

Summing up, achieving better estimates of dislocation velocities under extreme conditions will impact the planning and design of future experiments, where plastic relaxation depends on the velocity that dislocations can reach during loading. Phonon drag coefficients can also be inferred from the methodology presented here. An accurate determination of such damping coefficients is very much needed for advances in other simulation techniques such as discrete dislocation plasticity^26^. An example can be found in the recent contribution by Gurrutxaga-Lerma *et al.*^27^. It pointed out the unsuitability of two dimensional discrete dislocation plasticity to treat high strain rate processes and introduced a new formulation termed dynamic discrete dislocation plasticity (D3P) that is well-posed for the simulation of very high strain rate processes (10^6^ s^−1^ or more). Whilst a promising line of research, the authors highlight the need to accurately estimate damping coefficients in order to reduce uncertainties, and the method proposed in this letter can provide those coefficients.

In addition, knowledge of dislocation properties under such conditions would help inform designs of materials needed to perform under extreme conditions like those in nuclear reactors [ITER/NIF] or for space missions such as the Stardust mission.

Continuous advances in nanoindentation, laser technology and methods for generating ultra-fast shocks, and defect identification techniques, should make our proposed scenario available in the near future. Our model and simulations will motivate new experiments that might take this proposed scenario and extract information for realistic materials of technological interest.

## Methods

### Molecular dynamics simulation procedure

We have carried out MD simulations using LAMMPS^28^ to model Ta samples with up to 100 million atoms, but most of the simulations presented here are for ~20 million atom targets. A new embedded atom model (EAM) potential that describes high-pressure Ta properties very well^29^ is used. The potential was fit to match the experimental elastic constants (C_11_, C_12_, and C_44_ for cubic crystals) at zero pressure. We have calculated the elastic constants versus pressure up to 60 GPa, matching reasonably well with experimental results^30^. Further details on computational cost are given in the Supplementary Material. Detailed set-up description is presented below.

#### (a) Nanoindentation

Nanoindentation of bcc metals can lead to prismatic loop punching, according to our MD simulations^22^,^23^. Prismatic loops produced by nanoindentation are expected to be only 10–100 nm long. Conventional plasticity in bcc metals is typically associated with long screw dislocations, and not with prismatic loops, which could then be associated with nanoindentation for a well-annealed single crystal. These loops would not form junctions in single crystals, since they are sparsely emitted and there are no lattice defects like grain boundaries such as grain boundaries that could give rise to pile-ups. Indentation does produce near-surface defect structures, including extrusion of material on top of the surface. Prismatic loops would glide away and separate from this near surface region, and could be identified by High Resolution Transmission Electron Microscopy (HRTEM). An indenter with an effective radius of ~150 nm would lead to a plastic region with a depth of ~600 nm. We call this depth Z_1_. In this work, perfect (defect free) single crystal Ta samples with a top [001] surface were nanoindented with a stiff indenter, having a diameter of 10 nm, moving at 34 m/s. Unloading of the indenter led to surviving prismatic loops and no residual twinning, as shown in Fig. 1a. Indentation details are described elsewhere^22^. The prismatic loops are similar to those reported by Rudd^31^ for high-strain-rate tension and Tang *et al.*^19^ for homogeneous high-strain-rate compression of voids in Ta.

#### (b) Shock driving

We note that dislocation loop interaction with acoustic waves has been recently discussed: the loop acts only as a scattering center, and the amount of scattering could help to characterize dislocation densities^32^. Shock-dislocation loop interaction was also studied for fcc metals using MD simulations^11^. Laser-shocking Ta will lead to large dislocation densities below ~35 GPa, with twinning starting at that pressure, but only becoming prevalent above ~70 GPa, according to recent experiments^33^. Nanoregions of omega phase at pressures above ~50 GPa have also been observed^33^. The pressure range of 10–70 GPa, with strains up to ~10%, is of interest then to possibly produce detectable prismatic loops which could be driven supersonically. For W, strains of 5–10% were sufficient to lead to transonic and supersonic dislocations in simulations^16^. Comley *et al.*^7^ recently measured a flow stress of ~10 GPa for Ta at ~50 GPa. We note that, in shock physics, shock pressure typically refers to the longitudinal stress along the shock direction. A shock of 0.1 ns duration would move a prismatic loop emitted from an indented [001] surface about Z_2_ ~ Z_1_ + 200 nm, for an average velocity of 2 km/s, similar to expected transonic velocities in W^16^. In our simulations, before applying the shock, the top surface of the indented sample was removed, including pile-ups and most of the plastic region attached to the crater left by the indenter: only some prismatic loops, some dislocations at the center of the sample and some point defects survive. This simulates surface processing by EDM or other methods, and creates a flat surface, shown in Fig. 1b, which in turn would lead to a fairly flat and homogeneous shock front if a flat flyer was used. The remaining sample has dimensions 66 nm × 66 nm × 40 nm. Should surface processing turn out to be impractical in experiments, there is still no reason to believe that the proposed method cannot be implemented: imprints could lead to local non-planarity in the shock front but local variations would be averaged over the large cross-section, reducing uncertainties in shock planarity. To avoid residual stresses on the surface of the sample, the material was energetically minimized and then thermally relaxed. Periodic boundary conditions were applied in the transverse directions, and simulations were conducted at an initial temperature of 300 K. To apply a shock we set up a flyer-plate configuration, shown in Fig. 1(b). The flyer-plate has the same composition and crystalline orientation as the target, with a thickness of 20 nm (~4.5 million atoms). The flyer plate velocity was varied from 250 ms^−1^ to 1800 ms^−1^. These velocities translate into shock pressures in the range ~5-65 GPa, according to Ravelo *et al.*^29^. The shock wave traveling into the targets is extremely sharp, leading to strain rates of ~10^9^ s^−1^ for all the velocities studied here. Defects were tracked by means of Common Neighbor Analysis (CNA)^34^ and dislocations were identified by means of the dislocation extraction algorithm (DXA)^35^. Visualization was carried out using OVITO^36^.

### Wave speeds and dislocation velocity estimation

#### Wave speed estimation

The longitudinal wave travels with a velocity


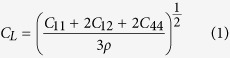


While the velocity of the transverse waves is


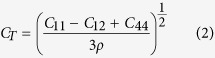


Eqs. (1) and (2) yield a longitudinal sound wave velocity 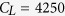 ms^−1^ and a transverse sound wave velocity 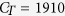 ms^−1^ at zero pressure. The longitudinal and transverse wave velocities were calculated by taking into consideration the variation of density with pressure, using the Hugoniot data for the potential^29^.

### Dislocation velocity estimation

Dislocation motion, growth and shape changes are a complex three dimensional (3D) process. Dislocation edge components were observed to glide exclusively in <111> direction and an effective 1D motion was identified. The dislocation velocity is inferred from the dislocation transit distance in this effective 1D direction divided by the transit time: V_d_ ~[(Z_2_ − Z_1_)/Δt]. The experiment is designed such that Z_2_ − Z_1_ is large compared to the uncertainty in Z_1_. In particular, given that dislocation loops would be situated at Z_1_ ~ 600 nm after a typical nanoindentation experiment, the plastic region would advance from Z_1_ ~ 600 nm to Z_2_ ~ 800 nm depth after a 0.1 ns flat-top shock, such as generated by the flyer plate in the MD simulation. For more typical pulse shapes, the applied shear stress behind the shock would not be a perfect flat top, and it would be sustained for 0.5–5.0 ns, depending on the details of the design. For a stress duration of 1 ns behind the shock front, the dislocation distance traveled, Z_2_, would increase to 1 um or more, which should be readily observable with current characterization techniques, such as TEM. In both the experiment and simulations, knowing the effective shock pulse duration and Z_1_ (with some uncertainty) and measuring Z_2_ allows the dislocation velocity to be inferred at high stress and strain rates as V_d_ ~[(Z_2_ − Z_1_)/Δt]. In addition, in the MD simulations it was possible to measure the instantaneous dislocation velocity along its direction of motion by tracking its displacements in time, similar to what has been done for fcc^16^ and bcc metals^19^. As a final note, in experimental setups the shock front does not build up instantaneously, but with a rise time inversely proportional to the strain rate^37^, as discussed in the Suppl. Mat. Our simulations correspond to rise times ranging from 0.1 ns to 10 ns, easily reached with state of the art laser shock facilities. However, current ultra fast VISAR (Velocity Interferometer System for Any Reflector) technology may face difficulties in the proper determination of the shock front profiles as strong shock conditions might lead to sub-nanosecond rise times, potentially introducing a source of uncertainty quantifiable in a experimental campaign.

## Additional Information

**How to cite this article**: Ruestes, C. J. *et al.* Probing the character of ultra-fast dislocations. *Sci. Rep.* **5**, 16892; doi: 10.1038/srep16892 (2015).

## Supplementary Material

Supplementary Information
