# Supplementary material for: Probing the character of ultra-fast dislocations
Source: Sci Rep. 2015 Nov 23;5:16892. doi: 10.1038/srep16892 (PMC4655350; doi:10.1038/srep16892)
Supplement: Supplementary Information [file srep16892-s1.pdf]

Supplementary Information

"Probing the character of ultra-fast dislocations" by C.J. Ruestes, E.M. Bringa, R.E. Rudd, B.A. Remington, T.P. Remington, M.A. Meyers

1- Computational cost of the simulations

We have used the LAMMPS MD code (http://lammps.sandia.gov/). LAMMPS is the acronym for Large-scale Atomic/Molecular Massively Parallel Simulator. The code was selected because: (i) achieves extremely good performance in large parallel computers (http://lammps.sandia.gov/bench.html#eam), and (ii) it is well tested and efficient for these large indentation [S1] and shock simulations [S2]. A many-body interatomic potential developed especially for shocked tantalum was used in order to give physically realistic results [S3]. Simulation runs were typically performed on 1024 processors at a state-of –the-art IBM BlueGeneQ computer at LLNL. The performance of LAMMPS over such architecture for the potential we used is of approximately 5 microseconds per atom /step/ core. Nanoindentation simulations were performed using an integration timestep of 1 fs and the process consisted of indenter insertion, hold and removal for a total simulation time of 0.5 nanosec., that is 500000 simulation steps performed on a 20 million atoms sample. Given the performance above yields a total simulation time of 16 hours, but those 16 hours do mean 16384 CPU-hours per run. A comprehensive set of simulations was performed testing indenter size and penetration velocity, sample temperature and orientation accounting for dozens of simulations and a total simulation time for the nanoindentation part of nearly 4e5 CPU-hours. A somewhat smaller computation time was used to perform the shock-compression simulations until the authors were confident of the results and proposed methodology. Therefore, we used for this study nearly 0.7 million CPU hours, without including CPU time for analysis.

2- MD applied to nanoindentation

Cold rolling or pre-tension can be used to add dislocation loops to a sample. However, they would not produce any localized plastic region, and we prefer to use nanoindentation to generate a well defined region with plasticity. The suitability of the MD technique for the study of nanoindentation plasticity is well documented, including its limitations. Even though there is certainly a gap in timescales between nanoindentation experiments and MD simulations, the latter has been successfully applied in elucidating plasticity mechanisms under the indenter. There are a number of papers that discuss potential problems when comparing MD simulations of indentation and related experiments. The authors of the current letter recently published two papers about nanoindentation of Ta [S1,S4]. In those contributions, we showed that MD was able to successfully reproduce pileup geometry, plastic anisotropy, etc. One of the authors, C.J. Ruestes, also co-authored a recent paper [S5] discussing the extension of the plastic zone under a nanoindenter, both in fcc and bcc metals, with results in agreement with finite element method calculations and experiments.

3- Preliminary experimental results

The experimental portion of the program has already been initiated with significant progress. We already reported TEM of single nanoindents in Remington et al. [S1], which was being refereed when this paper was submitted. Now we can report that nanoindentations were created in regular arrays and the dislocation structure under them was identified by transmission electron microscopy. This is shown in Figure FS1 below, which was generated for similar experimental conditions as described by Remington et al. The next stage is the quantification of the dislocation loop populations. It is important to emphasize that our experiments are being guided by computations. This first stage of the research, presented in the current manuscript, identified the mechanisms and predicts the distances to which the dislocations will travel when a shock is applied. The step of shocking nanoindented samples will be accomplished similarly to Ta shocks in pristine samples, as presented by [S6], using samples as the one in Fig. FS1a.

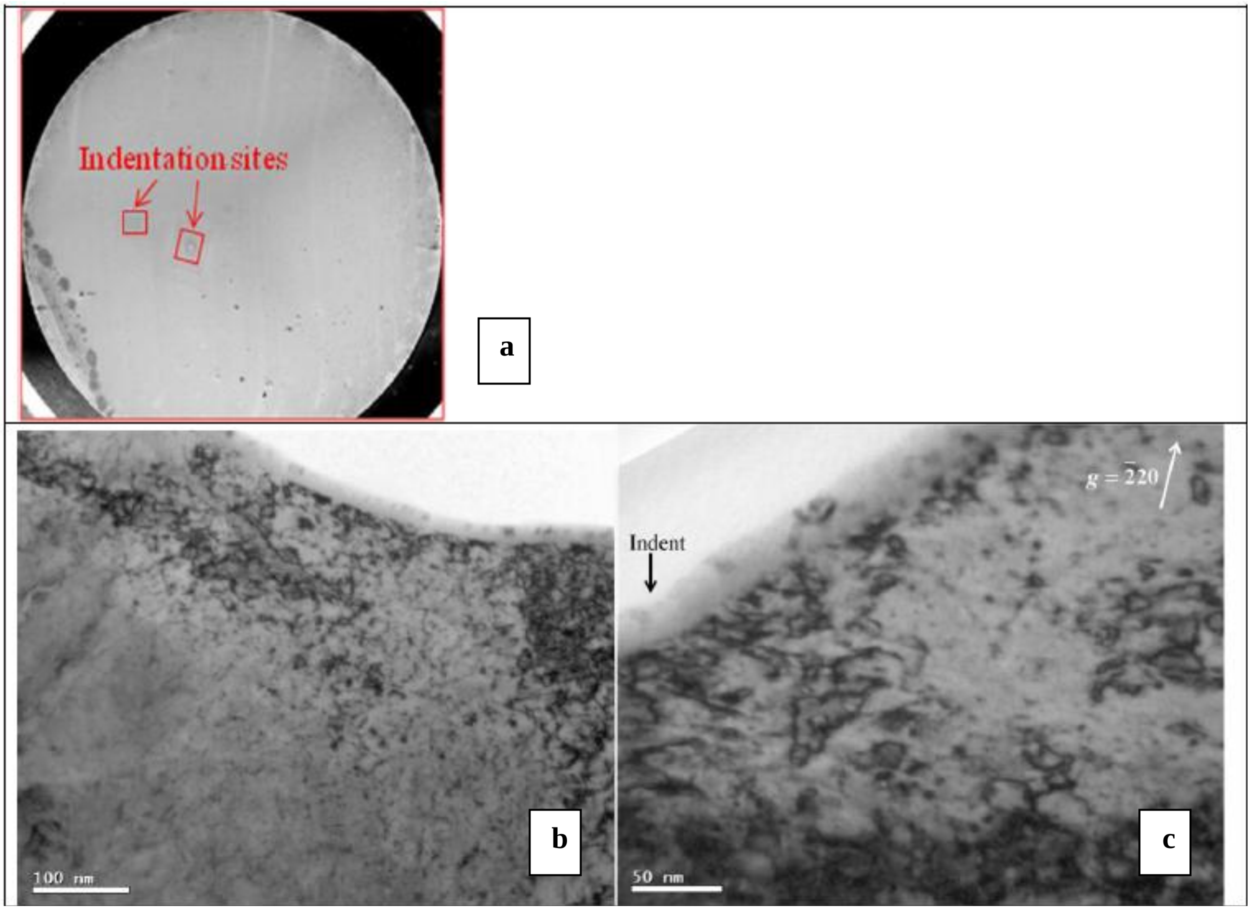

Figure FS1 (a) Nanoindentation sites in monocrystalline tantalum; (b, c) dislocation pattern around nanoindentation site imaged by transmission electron microscopy.

4- Discussion about possible loop diffusion and mobility

Radiation damage has been used in many studies to produce relatively localized plastic regions, containing small dislocation loops as a result of radiation-induced defects. In particular, W and Fe are materials of interest for the nuclear industry and have generated a large amount of research including what happens for small loops generated by radiation damage. Regretfully, this is not the case for Ta, and we have not been able to find any specific study on radiation damage and prismatic loop diffusion in Ta. However, we can compare mobility from individual dislocations and draw some conclusions.

Given the large amount of research on Fe, ubiquitous as it is of many technological applications, it could be a candidate material for our proposed experiments. However, we opted for Ta to pursue the present study due to several reasons in its favor. First of all the high Peierls stress barrier to dislocation motion plus the certainty that Ta maintains its bcc phase up to extremely high pressures and temperatures, while bcc Fe can undergo many phase transformations as pressure builds up. The stability of the bcc phase is guaranteed by the interatomic potential chosen.

Gilbert et al [S7] studied the stress and temperature dependence of screw dislocation mobility in alpha-Fe by molecular dynamics and obtained friction coefficients in the order of 2.3 to 2.9 exp(-4) Pa*s and dislocation velocities in the range of 600 to 1000 m/s for stresses in the range of 1 to 1.5 GPa. Queyreau et al [S8] studied edge dislocation mobility in bcc Fe obtained by molecular dynamics resulting in friction coefficients in the range of 1 to 3 exp(-4) Pa*s, consistent with viscous dynamics and dislocation velocities of up to 1300 m/s. In other words, bcc Ta exhibits a dislocation mobility one order of magnitude lower than the results for bcc Fe measured by the ratio of phonon drag coefficients, and this would significantly reduce loop motion as compared to Fe studies.

Another consideration is that our MD loops include about N=150-200 point defects (interstitials or vacancies). Loops punched out by a nanaindenter would include thousands of interstitials, reaching sizes much larger than those in radiation damage experiments and, given that mobility is size dependent, their mobility would be significantly reduced. According to the Dudarev et al [S9], dislocation loop mobility is expected to change as the inverse of the loop perimeter, and therefore as N^0.5, which is somewhat slower than the 0.8 exponent found by Arakawa et al. (Eq. 3 in [S10]). In addition, as pointed out by Derlet and co-workers, random-walk diffusion implies that ~0 [S11], Appendix and Fig. 5b, see also Arakawa Science figure 3a]. This means that, even if there is significant diffusion, the average location of the loops would remain somewhat localized. This can be seen in Arakawa's paper too, in Fig. 3A, and in Dudarev et al., [S9], Figs 2 and 3). Since we actually use the overall extent of the plastic zone to deduce dislocation velocities, a reduced level of diffusion by some individual loops would not be problematic.

A key aspect in this research is the selection of virtually defect free single crystals of the highest purity which is now explicitly mentioned in the manuscript. Still, point defects may hold the dislocations in place following nanoindentation and until the shock experiment, which would be a positive outcome for our proposal. When the shock arrives the defect atmosphere cannot

keep up with the fast dislocation. Shock stresses can easily exceed the yield point associated with Cottrell atmospheres. It is important that the point defects not impede the motion of the dislocations as they are driven by the shock stress, but otherwise pinning may be positive, given that it will hold loops in place before/after shock loading.

Arakawa and co-workers had shown that nanometer-sized prismatic loops may undergo onedimensional diffusion in the lack of driving stresses provided temperature is sufficiently high (450 K and above), and their study takes place in the timescale of seconds. There are several reasons why this is unlikely to matter in our proposed experiments:

I) While our simulations do have nanometer-sized prismatic loops close to the order of magnitude of Arakawa et al, the prismatic loops resulting in experimental nanoindentation would be of the order of a hundred nanometers, that is two order of magnitude above that of Arakawa et al.[S10] Linked to this scale difference, mobility of prismatic loops is size-dependent in a way such that the larger the loop the lower its mobility, as mentioned above.

II) The high mobility in Arakawa varies as exp(-E/kT), with an effective threshold temperature for mobility of 450 K, due to defect atmosphere dragging. Certainly spark-machining might lead to localized temperatures in the sample when preparing TEM samples. However, we propose to use FIB machining instead, which should not lead to high temperatures. Therefore, both the preparation of the sample by nanoindentation and the preparation of the shocked sample for TEM are tasks performed at room temperature, and in principle, free of the effect reported by Arakawa et al.

III) Shock compression experiments as proposed would only cause the temperature of the sample to be above an hypothetical threshold for loop diffusion for a few tens of nanoseconds after laser shock [S12].

IV) Fe in the Arakawa experiment [S10] had 99.998 % purity, and C was expected to reach only sub-ppm levels in weight. Despite this low concentration, C appeared to be extremely relevant, renormalizing (increasing) the effective energetic barrier for dislocation diffusion. In our case, as mentioned above, impurities would also increase the diffusion barrier, but they would be overcome by the immense stresses of the shock wave. Therefore, pinning impurities would reduce diffusive motion without affecting significantly the distance the dislocation travels due to the shock wave.

Quantitatively, for a 21 nm loop, Arakawa et al [S10] give a diffusivity of 2 nm2/s for T ~600 K. No values are given for lower T, presumably because the loop is immobile. For a planar loop, the number of interstitials involved would be proportional to diameter^2. For a 200 nm loop, then one would then have 100 times more defects, and the diffusivity would decrease roughly by a factor of 10 (taking into account the inverse perimeter dependence from Dudarev et al. If going to 300 K, the decrease would be a further 4-5 orders of magnitude. So we have a decrease in 5-6 orders of magnitude with respect to Arakawa's results in Fig. 4, even assuming similar mobility, which in fact are lower for Ta than for Fe.

As mentioned before, there is not much data for Ta. However, in the same way as for other BCC metals, impurities will limit the diffusion in stress-free samples by renormalizing energy barriers. For instance in Fe, a small naked loop has a barrier of 0.4 eV at low T, while the barrier is increased to 1.2 eV for the loop with a C atmosphere [S10]. These barriers can be overcome by the immense pressures in the strong shock waves discussed in the paper.

Finally, while we focus here on simulations for half a dozen shock pressures for clarity, tens of simulations were conducted in order to understand the parameters for a converged simulation and to ensure the robustness of the dislocation behavior. Several analysis techniques (CAN [S13], DXA [S14]) were used to understand the dislocation mechanisms sufficiently well to have confidence that they would be active in experiments.

5. Estimates of Shock Front Thickness

Experimental determination of strong shock front thickness is extremely challenging. Often, as a first approximation, the Swegle-Grady (SG) [S15] approach is considered. For tantalum SG reads [S16]:

$$\dot{\varepsilon} = 27.34 \cdot 10^{-36} \times P_{shock}^{4} = \frac{\varepsilon}{t}$$

where P_{shock} is in unit of [Pa] and $\dot{\varepsilon}$ in s^{-1}.

The Rankine-Hugoniot equation connecting pressure P to specific volume V :

$$P = \frac{C_0^2 (1 - V/V_0)}{V_0 [1 - S(1 - V/V_0)]^2}$$

where C_0 is the ambient sound speed, and S is an equation of state (EOS) parameter. Strain is related to volume through:

$$\varepsilon = \ln \frac{V}{V_0}$$

The pressure dependence on strain, determined from the Rankine-Hugoniot equations and equation of state can be expressed as,

$$P = \frac{C_0 (1 - e^{\varepsilon})}{V_0 [1 - S(1 - e^{\varepsilon})]^2}$$

The shock front thickness δ can be estimated by computing the product of the shock speed u_s and time t derived from the Swegle-Grady equation [S16]:

$$\delta = u_s t = u_s \frac{\varepsilon}{27.34 \cdot 10^{-36} \times P^4}$$

The latter equation allows estimating the shock front thickness based on the Rankine-Hugoniot equation, the equation of state and the relationship between pressure and strain rate.

MD simulations of strong shocks in Ta display extremely sharp shock fronts [S17]. Experiments of laser shocks in metal thin films had revealed rise times of the order of 10 ps [S18,S19].

REFERENCES

[S1] Remington, T. P., et al. "Plastic deformation in nanoindentation of tantalum: A new mechanism for prismatic loop formation." Acta Materialia 78 (2014): 378-393.

[S2] Tramontina, Diego, et al. "Molecular dynamics simulations of shock-induced plasticity in tantalum." High Energy Density Physics 10 (2014): 9-15.

[S3] Ravelo, R., et al. "Shock-induced plasticity in tantalum single crystals: Interatomic potentials and large-scale molecular-dynamics simulations." Physical Review B 88.13 (2013): 134101.

[S4] Ruestes, C. J., et al. "Atomistic simulation of tantalum nanoindentation: Effects of indenter diameter, penetration velocity, and interatomic potentials on defect mechanisms and evolution." Materials Science and Engineering: A 613 (2014): 390-403.

[S5] Gao, Yu, et al. "Comparative simulation study of the structure of the plastic zone produced by nanoindentation." Journal of the Mechanics and Physics of Solids 75 (2015): 58-75.

[S6] Lu, Chia-Hui, et al. "Laser compression of nanocrystalline tantalum." Acta Materialia 61.20 (2013): 7767-7780.

[S7] Gilbert, M. R., S. Queyreau, and J. Marian. "Stress and temperature dependence of screw dislocation mobility in α-Fe by molecular dynamics." Physical Review B 84.17 (2011): 174103.

[S8] Queyreau, S., et al. "Edge dislocation mobilities in bcc Fe obtained by molecular dynamics." Physical Review B 84.6 (2011): 064106.

[S9] Dudarev, S. L., et al. "Langevin model for real-time Brownian dynamics of interacting nanodefects in irradiated metals." Physical Review B 81.22 (2010): 224107.

[S10] Arakawa, Kazuto, et al. "Observation of the one-dimensional diffusion of nanometer-sized dislocation loops." Science 318.5852 (2007): 956-959.

[S11] Derlet, P. M., M. R. Gilbert, and S. L. Dudarev. "Simulating dislocation loop internal dynamics and collective diffusion using stochastic differential equations." Physical Review B 84.13 (2011): 134109.

[S12] Lu, Chia-Hui, et al. "Laser compression of monocrystalline tantalum." Acta Materialia 60.19 (2012): 6601-6620.

[S13] Faken, D., & Jónsson, H. Systematic analysis of local atomic structure combined with 3D computer graphics. Comp. Mat. Sci., 2(2), 279-286 (1994).

[S14] Stukowski, A. & Karsten A. Extracting dislocations and non-dislocation crystal defects from atomistic simulation data. Mod. Sim. Mat.Sci. Eng. 18.8: 085001 (2010).

[S15] Swegle, J. W., & Grady, D. E. Shock viscosity and the prediction of shock wave rise times. J. Appl. Phys. 58(2), 692-701 (1985).

[S16] Murr, L. E., Meyers, M. A., Niou, C. S., Chen, Y. J., Pappu, S., & Kennedy, C. Shock-induced deformation twinning in tantalum. Acta materialia, 45(1), 157-175 (1997).

[S17] Tramontina, D., Erhart, P., Germann, T., Hawreliak, J., Higginbotham, A., Park, N., ... & Bringa, E. Molecular dynamics simulations of shock-induced plasticity in tantalum. High Energy Density Physics, 10, 9-15 (2014).

[S18] Gahagan, K. T., Moore, D. S., Funk, D. J., Rabie, R. L., Buelow, S. J., & Nicholson, J. W. Measurement of shock wave rise times in metal thin films. Physical review letters, 85(15), 3205 (2000).
[S19] Crowhurst, J. C., Armstrong, M. R., Knight, K. B., Zaug, J. M., & Behymer, E. M. Invariance of the dissipative action at ultrahigh strain rates above the strong shock threshold. Physical review letters, 107(14), 144302 (2011).
